# Mobile Health Projects in a High-Complexity Reference Hospital: Case Study

**DOI:** 10.2196/16247

**Published:** 2020-01-31

**Authors:** Inmaculada Grau-Corral, Margarida Jansà, Pau Gascon, Raimundo Lozano-Rubí, Percy Efrain Pantoja, Daria Roca, Valentín Aragunde Miguens, Diego Hidalgo-Mazzei, Joan Escarrabill

**Affiliations:** 1 Hospital Clinic de Barcelona Barcelona Spain; 2 Fundación Internet Salud y Sociedad Barcelona Spain; 3 Institut d'Investigacions Biomèdiques August Pi i Sunyer Grup de Recerca transversal en Atenció Primària Barcelona Spain; 4 Universitat Oberta de Catalunya PsiNET Barcelona Spain; 5 Universitat de Barcelona Barcelona Spain; 6 Instituto de Salud Global Barcelona Spain; 7 Consorci d'Atenció Primària Casanova Consorci d'Atenció Primària de Salut de l'Eixample (CAPSBE) Barcelona Spain

**Keywords:** mobile health, observational study

## Abstract

**Background:**

The widespread adoption of mobile and wearable devices and apps makes it essential to assess their possible impact on the management of health and diseases. Health care providers (HCPs) find themselves faced with a new situation in their setting with the proliferation of mobile health (mHealth) intervention tests. Few studies have addressed the development of mHealth and the methodologies to manage these apps in a tertiary hospital.

**Objective:**

The aim of this study was to evaluate the mHealth projects implemented in the Hospital Clínic of Barcelona to increase awareness of the context in which they are used and to develop policies for the development of good practice in mHealth innovation.

**Methods:**

A prospective, descriptive cross-sectional study was conducted in a highly specialized university hospital with 850 beds for adults and a reference population of 520,000 inhabitants. A specific questionnaire was developed based on the Mobile Health 5 Dimensions European (MOHE 5D-EU) theoretical model to find mHealth projects. Apps, telemedicine, and wearable devices were included in the systematic search. For that purpose, a vertical (top-down) email-based *snowball* process was conducted. Data were collected from February to December 2018 by conducting personal interviews with HCPs using a structured questionnaire.

**Results:**

During the study period, 45 interviews were conducted; 35 mHealth initiatives were found, with 25 targeted to patients and 10 to health professionals. Most mHealth initiatives (34/35, 97%) were related to the software field (apps and telemedicine initiatives), and one was related to wearable devices. Among the projects, 68% (24/35) were classified as medical devices or developments at the edge (developments susceptible to limitations depending on the *intended* use). In relation to data protection, 27 initiatives managing personal data (27/35, 77%) considered data protection legislation. Only 9% (3/35) of the initiatives had foreseen the use of interconnectivity standards. Most of the initiatives were funded by grants (14/35, 40%), sponsorships (5/35, 14%), or the hospital itself (5/35, 14%). In terms of clinical management, most projects were developed in the field of research, followed by professional tools, clinical information, and therapeutic education. Only 6 projects were involved with health care; all were led by either the industry or small and medium enterprises.

**Conclusions:**

This study helped create the design of a map of the mHealth projects conducted in our hospital that showed the stages of development of the different ongoing projects. This will allow monitoring of mHealth projects and construction of tools to reinforce areas with detected deficiencies. Our theoretical approach using a modified MOHE 5D-EU model was found to be useful for analyzing the characteristics of mHealth projects.

## Introduction

### Background

There are more than 5 billion mobile phone lines in the world [1,2]. Since the launch of the Google Play and iTunes platforms in 2008, the number of apps for mobile phones has continuously grown, exceeding 2.1 million for the Android platform and 1.8 million for the Apple platform in 2019 [3].

It has been estimated that there are about 325,000 apps [4] classified in the categories of health and medicine. Of these, 65% are dedicated to well-being (exercise, healthy lifestyle, control of stress, dieting, and nutrition). Those dedicated to specific diseases constitute 9% of the total, followed by those related to pregnancy (7%) and apps focused on treatment adherence or alarms to take medication (6%).

The widespread usage of mobile devices, apps, and wearable devices makes it essential to assess their possible impact on the management of health and diseases and to determine how health care providers (HCPs) manage their implementation. The mobile health (mHealth) care survey by Spok [5] (with more than 300 health care professionals, of which 44% were clinicians) found that more than half of the hospitals reported having mobile strategies in place (mostly telemedicine). The National Institute for Health and Care Excellence developed a document that describes an *evidence standards framework* for digital health technologies for English National Health Service providers in 2018 [6]. The World Health Organization also developed a guideline with recommendations for digital interventions aimed at *the health system strengthening* [7].

Technology providers and their organizations often monitor the market through studies such as the *Mobile Strategies in Healthcare report* by Spok, the report by the IQVIA Institute (formerly the Intercontinental Medical Statistics Health Institute), or the *Use, Evidence and Remaining Barriers to Mainstream Acceptance Patient Adoption of mHealth* [8], or they take a very simple approach such as Healthcare Information and Management Systems Society, with the classification of hospitals according to the *Electronic Medical Record Adoption Model* adoption scale [9].

HCPs are progressively integrating management and monitoring of the development of new mHealth interventions into ethics committee procedures. There are only a few studies that address the development of apps in a tertiary hospital [10].

To facilitate the management of knowledge related to mHealth, an observatory was created at the Hospital Clínic of Barcelona (HCB). The mHealth observatory has a technical committee comprising representatives from the different departments of the linked hospital, including information systems management, medical informatics, legal department, innovation, technology transfer, bioengineering, and patient experience. A coordinator directs a community of practice to ensure proper knowledge management within the organization. The functions of the observatory are to generate usable knowledge, facilitate the use of this knowledge, and manage the changes incurred [11].

The mHealth observatory has three subobjectives: first, the marshaling function, which refers to the collection of relevant datasets from various sources; second, the analysis and synthesis function regarding the transformation of data into usable knowledge and the integration of information to generate comprehensive understanding; and finally, the sharing function, which is related to facilitating the exchange of information to support decision making.

The first step is to be aware of the actual situation of the hospital and its environment. Therefore, the main objective of this study was to describe and map the mHealth projects implemented in a high-complexity reference hospital.

### Objective

The aim of this study was to identify and to describe the level of development of mHealth projects in the HCB and, thus, contribute to the improvement of mHealth policies in this health care center.

## Methods

### Study Design and Setting

A cross-sectional study was conducted in the HCB, a highly specialized hospital with 850 beds for adults and a reference population of 520,000 inhabitants.

An interdisciplinary team of researchers comprising two nurse educators, a specialist in patient experience, two specialists in public health, a general practitioner, two experts in medical technology, and a psychiatrist were commissioned to map the mHealth projects at the HCB.

In addition to focusing on obtaining concrete clinical or quality-of-life–related outcomes, the following indicators were also considered to determine the level of maturity of the projects:

- Assessment by the development team on whether the project results were medical devices (MDs) or not [12]
- Use of data and protection of the data [13] and the possible connection with health providers and electronic medical records [14-16]
- Development of viability or business plan to ensure the survival and scalability of the project [17]
- Patient participation

### Methodology

To complete the cartography of mHealth projects, a work plan was agreed upon by the research team. This included reaching a consensus as to the basic definitions of the research subject, choosing the theoretical approximation model, constructing an ad hoc questionnaire, and, finally, conducting the fieldwork.

A standard description of mHealth was adopted by the researchers: “mHealth are tools and strategies to improve health through the use of mobile technologies.”

*The inclusion criteria were as follows:* projects involving the participation of HCPs from the hospital and projects involving apps (autonomous software initiatives), telemedicine projects including the use of mobile devices, and projects with wearable devices (such as bracelet pedometers and glucose meters).

*The exclusion criteria were as follows:* ideas or projects with protocols that were inadequate to apply for competitive funding and projects developed without HCP participation.

The questionnaire was developed to collect contextual data and characterize the initiatives. It had 8 sections to characterize the ongoing initiatives and their level of development. Overall, 3 sections were related to affiliation, the use of mHealth in the setting of the interviewee, and a semistructured interview. The remaining 5 sections were related to the description of the project according to the Mobile Health 5 Dimensions European (MOHE 5D-EU) model, a theoretical reference model [11]. The questionnaire was developed considering five dimensions [18]: legal, technological, sustainability, patient participation, and clinical management:

1. The legal dimension had three sections. One section was related to whether or not the apps or the wearable devices were in fact MDs according to European and Spanish regulations. This dimension answers the questions “What is it?” The second section answers the question “Who is the owner?” (Who owns the software or the device related to intellectual property?). The last section was related to concerns about data protection. The definition of MD was as follows: a device is considered an MD if (1) it is a computer program (not an informational document only), (2) it performs some action on data (not just stored) to facilitate the interpretative tasks of the health personnel, (3) it benefits individual patients (no population aggregation and generic diagnostics according to literature), and (4) this is done for the purpose of care, provided in the definition of MD [12].
2. The technological dimension was related to interoperability, use of connection technology standards, and data management and data protection.
3. Sustainability was evaluated by the Research2Guidance models [17]. To classify the development phase, five categories were defined: project, when a project document of requirements was already available; in development, when a project was under construction; prototype, when the development of the project was complete but had not yet been tested with patients; pilot, when the test was conducted with patients; and working, when the project was already in use in the workplace.
4. A two-question approach was implemented in the patient dimension in relation to whether patients had been considered during project development. Project coordinators would be asked to confirm patient participation.
5. Finally, four categories were defined in the dimension of clinical management: one for HCPs and three for patients (research, assistance, and information or patient education).

To identify mHealth initiatives, a vertical (top-down) email-based *snowball* process was conducted. Once the coordinators of the initiatives were identified, they were asked to attend an interview conducted by a researcher using the related questionnaire.

The data were collected by personal interviews with HCPs over a 6-month period in 2018.

The research team classified the projects by medical specialty and the theoretical strategy followed and incorporated the projects in a surveillance system using five-dimensional characterization.

The app type classification used by the Mobile App Rating Scale (developed by the Queensland University of Technology and the Young and Well Cooperative Research Centre) [19] was chosen to label the type of apps for patients to identify the use of the underlying theoretical strategies of the projects for patients. The following aspects were used in different projects: evaluation; feedback, information/education, and monitoring/follow-up; goal setting; counseling/guidelines/skills training; cognitive behavioral therapy (CBT)–behavioral (positive events); CBT–cognitive (thought challenging); acceptance and commitment therapy; mindfulness/meditation, relaxation, and gratitude; strengths; and others.

In addition, the Fogg triad [20] was used to classify professional apps as a tool, mediator, or social actor.

#### Statistical Analysis

The analysis of power and statistical significance used in studies that test a hypothesis do not apply in descriptive studies such as this. Therefore, the responses of the questionnaires were summarized using descriptive statistical techniques such as mean for continuous data, median for stratified data, and percentage as needed. The answers were also examined graphically, with percentiles to identify outliers.

#### Ethical Aspects

Data collection is one of the functions of the mHealth observatory. The observatory was implemented by the administration of the hospital as part of an innovation strategy. No data were collected from patients. All the participant projects are linked to an HCP of the hospital.

## Results

During the 6-month study period, 45 mHealth projects were identified; 35 met the inclusion criteria, of which 25 were targeted to patients and 10 were related to HCPs. A total of 34 projects were in the software field (apps and telemedicine initiatives), and only 1 was in the wearable devices field (a fall detector).

With regard to mHealth projects, 8 projects were related to infectious diseases, mainly drug dose calculators; 7 were related to mental health; 2 projects each were related to central services neonatology, cardiology, nephrology, pneumology, anesthesia, and emergency care; and 1 project each was conducted in pharmacy, endocrinology, oncology, rheumatology, gastroenterology, and obstetrics (Table 1).

With reference to theoretical background/strategies, only 8 of the 14 possible categories of apps classification were used to categorize patient apps, and only 2 of 3 Fogg categories were used for professional apps. The results obtained are shown in Table 2.

The results of the questionnaire conducted during the interviews were codified in a calculation sheet, and the results were presented in the five dimensions of the MOHE 5D-EU model (Table 3).

**Table 1:** Summary of mobile health initiatives found according to the medical specialty.

Focus of the mobile health initiatives	Projects, n
Infectious diseases	8
Mental health	7
Central services	2
Neonatology	2
Cardiology	2
Pneumology	2
Nephrology	2
Anesthesia	2
Emergency	2
Pharmacy	1
Diabetes	1
Oncology	1
Rheumatology	1
Gastroenterology	1
Obstetrics	1

**Table 2:** Theoretical background and strategies (total number of mobile health initiatives=35).

Mobile health initiatives, Characteristics		Values, n (%)^a^
**Patients (n=25)**		
	Feedback	16 (64)
	Monitoring/tracking	15 (60)
	Information/education	13 (52)
	Advice/tips/skill training	11 (44)
	Assessment	5 (20)
	Goal setting	2 (8)
	CBT^b^–behavioral positive events	1 (4)
	CBT–cognitive thought challenging	1 (4)
**Professionals (n=10)**		
	Tool—increases capability	9 (90)
	Medium—provides experience	2 (20)

^a^Note that one app can have more than one characteristic.

^b^CBT: cognitive behavioral therapy.

**Table 3:** Legal, technological, sustainability, and patient participation aspects adapted from the Mobile Health 5 Dimensions European model.

Dimension, Detail				Patients, n		Professionals, n		Total, n (%)	
**Legal**									
	**What is it?**								
			Medical device		8		5		13 (37)
			Edge		10		1		11 (31)
			Nonmedical device		6		2		8 (23)
			Telemedicine		2		1		3 (9)
	**Who is the owner?**								
			Hospital		3		2		5 (14)
			Hospital+public/nonprofit		7		1		8 (23)
			Hospital+private		2		6		8 (23)
			Hospital+public/nonprofit+private		6		1		7 (20)
			No hospital		7		0		7 (20)
**Technological**									
	**Willingness to connect with the hospital system**								
			Yes		11		2		13 (37)
			No		14		8		22 (63)
	**Uses interoperability standards**								
			Yes		3		0		3 (9)
			No		13		7		20 (57)
			Telemedicine		2		1		3 (9)
			Don’t know or no opinion		7		2		9 (26)
**Sustainability and phase**									
	**Paid by the user**								
			Pay per download		0		2		2 (6)
			In-app purchase		3		0		3 (9)
			License		0		2		2 (6)
			Crowdfunding		1		1		2 (6)
			Linked to a medical device		2		0		2 (6)
	**Paid by a third party**								
			Grants		12		2		14 (40)
			Sponsorships and donations		4		1		5 (14)
			Institutions		4		0		4 (11)
			Software as Service		2		0		2 (6)
	**Paid by the hospital**								
			Hospital Clínic		3		2		5 (14)
	**Phase**								
			Project		4		1		5 (14)
			Development		0		2		2 (6)
			Prototype		5		1		6 (176)
			Pilot		7		1		8 (23)
			Working		8		5		13 (37)
			Discarded		1		0		1 (3)
**Patients**									
	**Focus of projects**								
			Professional		N/A^a^		10		10 (29)
			Patients		25		N/A		25 (71)
	**Patient participation in projects**								
			Continued		14		N/A		14 (56)
			Punctual		6		N/A		6 (24)
			They have not participated		5		N/A		5 (20)
**Clinical management**									
	**Area**								
			Information and patient education		6		0		6 (17)
			Health care		7		0		7 (20)
			Research		12		0		12 (34)
			Professionals		0		10		10 (29)

^a^Not applicable.

### Legal Dimension

In this dimension, the characteristics of the projects were evaluated. In addition to the 3 basic categories, *apps*, *telemedicine*, and *wearable devices*, it was necessary to determine whether developments could be classified as MD or not, considering that the malfunction of such a device may affect people’s health. The results were as follows: 13 developments were classified as *MDs*, 11 were at the *edge* (developments susceptible to limitations depending on the *intended use*), 8 projects were classified as *non-MDs* (eg, *Clinic Maps*), and 3 were classified as *telemedicine* (Table 3 under *Legal*).

The ownership of the development in response to the question of “Who’s the owner?” was also analyzed in terms of responsibility, profits, and use of the brand. Of the total, 8 initiatives were found to be public (including 5 from the hospital) and 27 were from private entities.

With regard to data protection and considering that the whole sample comprised apps that do not collect sensitive data, a notable 77% (27/35) of the total developments (all sensitive cases) had foreseen data protection, thereby indicating a high level of awareness toward this topic.

### Technological Dimension

In the technological dimension, it was important to determine whether the use of connection standards was foreseen (interoperability) as this would allow data sharing from the apps to the hospital information systems, which would contribute to knowledge building. Similarly, it was determined whether the connection between apps and the hospital information systems was planned.

Only 9% (3/35) of the initiatives had foreseen the use of connection standards, and 26% (9/35) were not aware of these standards. With regard to the projection to connect with the hospital information systems, a little over one-third (13/35, 37%) of the project coordinators aimed for full integration (Table 3, under *Technological*).

### Economic Management Dimension (Sustainability and Phase)

The section on economic management included the business model or the prognosis of sustainability and the phase of the development cycle in which the projects were located in the cycle (Table 3 under *Sustainability and phase*).

The research team added two categories to the project financing model (crowd funding and hospital funding). The results were largely dominated by scholarships, grants, and sponsorships, which places the developments at low levels of business. Note that a project could have more than one funding source.

The lack of a business plan beyond the scholarships was of note. Having such a plan would facilitate the pathway to the market. Sponsorship and hospital funding were also determined to be the usual sources of funding.

Overall, 5 categories were described to classify the development phase: project, in development, prototype, pilot, and working.

The projects were found to be spread across all phases of the development cycle, with most projects in the working phase (n=14), followed by those in the *pilot* phase (n=8).

### Patient Dimension

In relation to the dimension of patient participation, 20 projects responded positively to the question about the involvement of patients. Considering that 10 projects were aimed at HCPs and 2 projects at information about the operation of the hospital, the percentage obtained was very satisfactory. Overall, 80% (20/25) of patient participation was on an ongoing basis. On the basis of the information collected, it was not clear whether patient participation had taken place from the beginning or not.

### Clinical Management Dimension

The dimension of clinical management provided interesting insights into the models of project creation. This section was divided into four categories *information and patient education*, *health care*, *research*, and *professional*, and projects were able to move from one category to another over time. An app was considered *professional* when the patient did not interact directly with the app.

Most projects were in the category of research (n=12) and were usually initiated by clinicians to solve problems associated with patient care. Tools aimed at helping achieve diagnoses predominated in the category of *professional* (n=10). In the *information and patient education* category (n=6), various initiatives by small entities were found. In addition, the industry also had an important role in health care initiatives within the field of industry products (n=7), with apps related to enabling the follow-up of patients with a product marketed by their company.

## Discussion

### Principal Findings

The results of this study show that it is possible to generate usable knowledge related to the collection of relevant datasets. Analysis and synthesis functions were also achieved, transforming data into practical knowledge and integrating the information to generate a comprehensive understanding. In addition, the application of the MOHE-5D-EU contextual model with the five dimensions used in this study, using an adapted questionnaire, proved to be useful in characterizing mHealth projects.

In the projects reviewed, a fairly established accomplishment of data protection rules and patients’ participation was found (although not always in a structured way).

Knowledge related to the development of new projects should be potentiated to help determine whether or not an initiative is to be used as an *MD* and to evaluate sustainability, the importance of the use of technological standards to facilitate connectivity, and in relation to the exploitation of data, among other aspects.

To know if a development will be an MD is important, especially considering that 68.5% of the projects found are likely to be MDs and that the new, more restrictive European regulations will be effective from May 2020 [12]. Although it should be noted that the developments led by hospital professionals are mostly in the research quadrant, where the MD commercialization regulations are not yet applicable, they will be mandatory as soon as the development enters the market. Moreover, making proposals solely in the domain of research, without considering sustainability and, therefore, its release to the market, punishes the consolidation of projects.

Unawareness of interoperability standards and their great importance for the exchange of health information and the construction of knowledge significantly reduced the contribution that mHealth can provide. It does not allow HCPs access to systematic collection of data, leaving the data fractionated in different platforms and of no benefit to electronic medical records.

One strength of this study was that it provided an overall vision of mHealth in our institution. By categorizing the results, the research team was able to develop a map of the projects and observe their weaknesses and strengths, which subsequently allowed the team to develop a toolkit to help new initiatives and those which are ongoing.

One limitation of the study was the introduction of aspects of different disciplines (eg, legal, information technology, and human behavior). It is more complex to analyze an interdisciplinary setting.

Funding of highly personalized initiatives through grants makes it difficult to detect all the initiatives undertaken. In these cases, the Ethics Committee of Investigation is the gatekeeper.

In addition, it was difficult to establish comparisons with previous studies as the studies available usually focus on the content of an initiative and on achieving a concrete, clinical and/or quality-of-life outcome [10,19,21,22] rather than context viability.

The mHealth observatory proposed the development of a series of guidelines and tools to facilitate the adequate development and implementation of useful, advantageous, and sustainable mHealth projects.[22]

Studies designed to link mHealth to the diagnosis and improvement of patients’ experiences would be of interest to define the scenarios that patients face.

### Conclusions

A map of the mHealth projects available in the HCB was created within a complex scenario involving different approaches and many different professionals, with many of the projects being developed without sufficient multidisciplinary knowledge.

From now on, we will be able to monitor the development of mHealth projects within the hospital and build tools that assist the reinforcement of the detected deficiencies.

Finally, according to the results obtained, our theoretical approximation model, which was an adaptation of the MOHE 5D-EU model, was found to be useful for analyzing the characteristics of mHealth initiatives and detecting their strengths and weaknesses.

## Abbreviations

CBT: cognitive behavioral therapy
HCB: Hospital Clínic of Barcelona
HCP: health care provider
MD: medical device
mHealth: mobile health
MOHE 5D-EU: Mobile Health 5 Dimensions European
